# Literature review of myasthenia gravis complicated by myocardial damage

**DOI:** 10.1097/MD.0000000000050927

**Published:** 2026-10-02

**Authors:** Chaofang Lei, Jiaxu Chen, Zhigang Chen, Le Xie, Dahua Wu, Junlin Jiang

**Affiliations:** a Department of Neurology, Beijing Tiantan Hospital, Capital Medical University, Beijing, China; b Department of Neurology, Hunan Provincial Hospital of Integrated Traditional Chinese and Western Medicine (The Affiliated Hospital of Hunan Academy of Traditional Chinese Medicine), Changsha, China; c School of Traditional Chinese Medicine, Beijing University of Chinese Medicine, Beijing, China; d Department of Neurology, Dongfang Hospital, Beijing University of Chinese Medicine, Beijing, China.

**Keywords:** AChR antibody, anti-titin antibody, literature review, myasthenia gravis, myocardial damage, myocarditis

## Abstract

**Background:**

Myasthenia gravis (MG) is a chronic autoimmune disorder predominantly driven by pathogenic autoantibodies directed against postsynaptic acetylcholine receptors, impairing neuromuscular transmission. Although cardiovascular complications remain relatively uncommon among MG patients, their emergence markedly elevates the risk of fatal outcomes. This review synthesizes existing evidence to delineate the clinical manifestations of MG-associated myocardial damage, offering clinicians a comprehensive framework for early detection and evidence-based therapeutic intervention.

**Methods:**

We analyzed 84 cases of myasthenia gravis associated with myocardial damage from the database and comprehensively evaluated their clinical characteristics.

**Results:**

The average age of patients presenting with myasthenia gravis and concurrent myocardial damage was 64.5 years. Cardiac complications predominantly included myocarditis, takotsubo cardiomyopathy, acute myocardial infarction, and coronary spastic angina. Serological analysis revealed 2 principal autoantibodies: acetylcholine receptor (AChR) antibody and anti-titin antibody. Clinical outcomes demonstrated a 72.6% survival rate alongside a 23.8% case fatality rate. These variables were more frequently observed among fatal cases but were not statistically associated with mortality: AChR antibody positivity, thymoma, myocarditis, immune checkpoint inhibitor use, and acute myocardial infarction.

**Conclusion:**

This study represents the inaugural comprehensive analysis examining the association between myasthenia gravis and concurrent cardiac injury. Our research elucidates potential pathophysiological pathways underlying this clinical entity. These findings highlight the need for clinical awareness and screening of myocardial involvement in MG patients and may provide preliminary evidence for future consensus development.

## 1. Introduction

Myasthenia gravis (MG) is an autoimmune disorder characterized by pathogenic autoantibodies impairing neuromuscular transmission, resulting in generalized skeletal muscle weakness. The hallmark clinical feature is fluctuating fatigability, typically exhibiting diurnal variation with milder symptoms in the morning that progressively worsen throughout the afternoon. Epidemiologically, MG demonstrates an incidence rate ranging from 4.1 to 30 cases per million person-years, while prevalence estimates vary between 150 and 200 cases per million population.^[1]^ In China, the reported incidence approximates 0.68 cases per 1,00,000 individuals. While MG may manifest at any age, its bimodal age distribution predominantly affects young women (20–39 years) and elderly men (50–70 years).^[2]^

The condition carries significant morbidity, with an in-hospital mortality rate of 14.69‰, primarily attributable to respiratory complications, including failure and pulmonary infections.^[3]^ Emerging evidence suggests that MG-associated myocarditis shares similar autoimmune pathogenesis. Despite its high fatality rate, contemporary therapeutic approaches can achieve disease remission, underscoring the importance of early clinical recognition and vigilant monitoring for cardiovascular complications in MG patients.

This comprehensive review systematically examines published cases of MG with concomitant myocardial damage, analyzing potential mechanisms underlying cardiovascular adverse events to enhance our understanding of this critical disease manifestation.

## 2. Materials and methods

### 2.1. Literature retrieval and statistical analysis

We comprehensively searched multiple databases, including PubMed, Web of Science, China National Knowledge Infrastructure (CNKI), VIP Database, and Wanfang Data. According to the description of myocardial damage in the included literature, myocardial damage includes the following types: myocardial damage, myocardial damage, myocarditis, myocardial infarction, myocardial ischemia, coronary artery spasm, arrhythmia, arrhythmology, heart failure, sudden cardiac death, stress-induced cardiomyopathy, broken heart syndrome, takotsubo cardiomyopathy.

Our literature review encompassed all available records in these databases through December 2024. Analyses were performed using SPSS version 20.0 (IBM Corp, Armonk). The complete search strategy for the database is shown in Table 1.

**Table 1:** Search strategy.

Key concept #1	Key concept #2		Possible additional keywords	Date range	Limiters
	AND	“myocardial injury” OR			
“myasthenia gravis” OR “myasthenic crisis”		“myocardial damage” OR “myocarditis” OR “myocardial infarction” OR “myocardial ischemia” OR “coronary artery spasm” OR “arrhythmia” OR “arrhythmology” OR “heart failure” OR “sudden cardiac death” OR “stress-induced cardiomyopathy” OR “broken heart syndrome” OR “takotsubo cardiomyopathy”	status indicators	Before December 2024	Language: English, Chinese Type of resource: journal articles

### 2.2. Inclusion criteria

Literature on the correlation between myasthenia gravis and myocardial damage; literature with a clear diagnosis of myasthenia gravis, where the patient is diagnosed with MG based on international consensus guidance for the management of myasthenia gravis; clearly stated and reported research on myocardial damage based on the international consensus guidelines for diagnosis; and randomized controlled studies, case reports, and case series.

### 2.3. Exclusion criteria

Studies without a clear diagnosis of myasthenia gravis; literature for which the full text cannot be obtained; republished literature; and conference abstracts and reviews.

### 2.4. Literature screening and data extraction

Literature screening process: We imported all studies obtained from the relevant databases into EndNote X9.1 software for duplicate checking and removal. We screened based on the title and abstract to select relevant research. We read the full text and screened the literature that met the requirements. Data extraction was carried out by 2 independent reviewers using the data extraction form. If there was a dispute over the results, a third-party review was conducted. Data extraction was based on the Joanna Briggs Institute (JBI) checklist (e.g., country, study setting/context, participant characteristics, outcome measures, and key findings). The Preferred Reporting Items for Systematic reviews and Meta-Analyses (PRISMA) flow diagram is shown in Figure 1.

**Figure 1.**
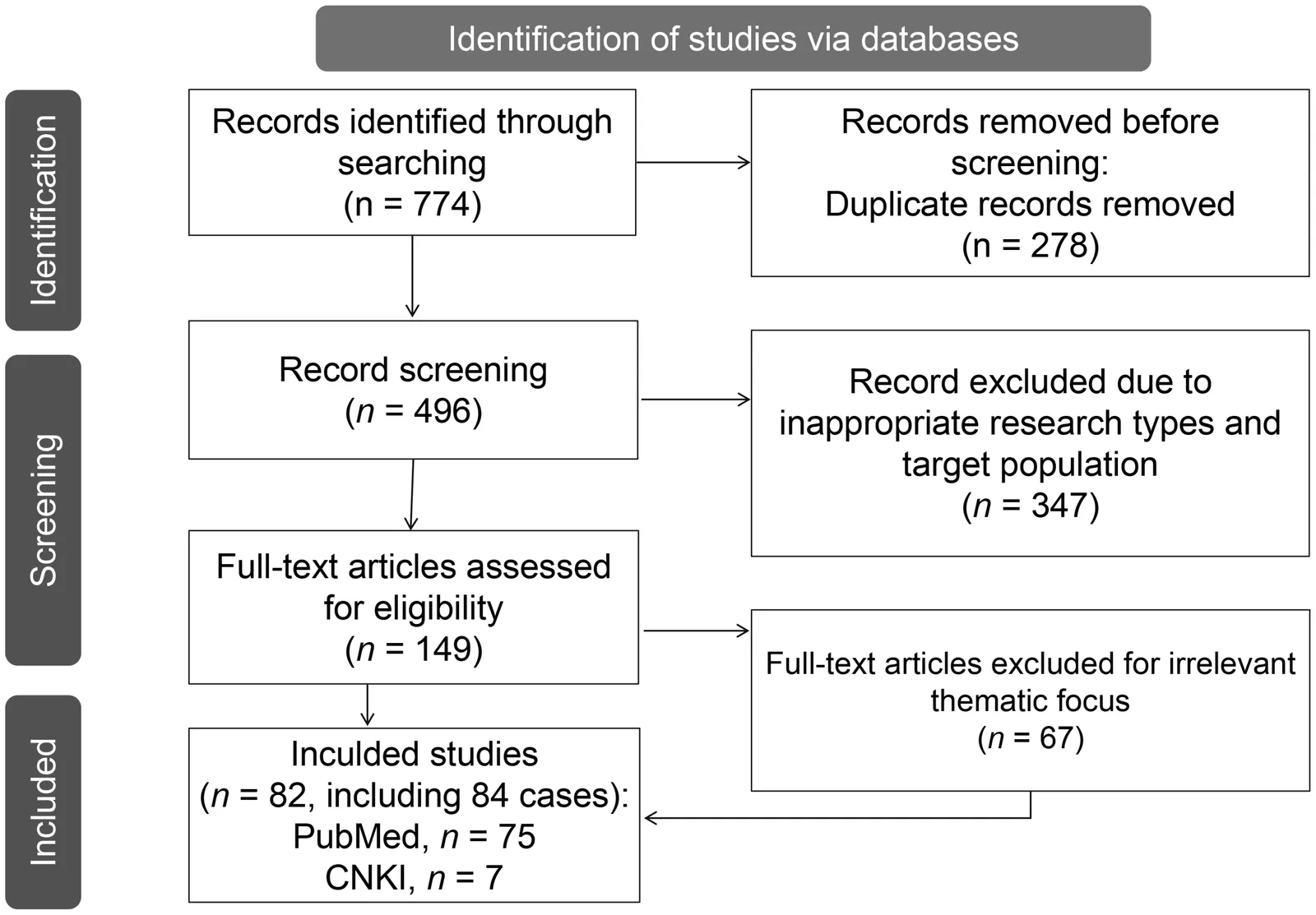
PRISMA flowchart. CNKI = China National Knowledge Infrastructure, PRISMA = Preferred Reporting Items for Systematic reviews and Meta-Analyses.

## 3. Results

### 3.1. Clinical characteristics

As presented in Table 2, we analyzed the demographic and clinical characteristics of patients diagnosed with MG complicated by myocardial damage in the database.^[4-85]^ The multinational cohort encompassed patients from 22 distinct geographical regions, exhibiting predominant representation from China (constituting 28.6%, n = 24/84), the United States (accounting for 21.4%, n = 18/84), and Japan (representing 19.0%, n = 16/84), alongside several Western European nations, notably France, the United Kingdom, and Spain. Figure 2A provides a comprehensive visualization of these case origins.

**Table 2:** Summary of MG cases with myocardial damage.

Study/date of publication	Nation	Sex	Age	Myocardial damage	Inducement	Pathogenic antibody	Thymus	Prognosis
1979	China	Female	31 yr	Heart failure	Thymotomy	/	Thymic hyperplasia	Rapidly improved 2 d
2022	China	Female	51 yr	Stress cardiomyopathy	Pulmonary infection	/	Thymoma	Resolved within 6–7 wk
2021	China	Female	47 yr	Myocarditis	/	/	/	improved 10 d
2011	China	Male	61 yr	Acute myocardial infarction	Influenza	/	/	Resolved within 2 wk
2023	China	Female	79 yr	Acute myocardial infarction	Pyridostigmine Bromide	AChR antibody, anti-titin antibody	Normal	Death
2022	China	Female	69 yr	Myocardial hypertrophy	Tacrolimus	/	/	In stable condition
2018	China	Female	68 yr	Acute myocardial infarction	Pulmonary infection	/	/	Resolved within 2–3 wk
2013	Greece	Female	71 yr	Non-coronary myocardial infarction	Pyridostigmine bromide	/	Thymoma	Death
2000	Japan	Female	71 yr	Coronary spastic angina	Anticholinesterase medication	Anti acetylcholine receptor antibody	Thymoma	Rapidly relieved
2005	Italy	Female	75 yr	Acute myocardial infarction	Anticholinesterase drugs	/	/	significant improvement
2004	Japan	Female	83 yr	“Takotsubo”-shaped cardiomyopathy	Plasmapheresis	/	Normal	Resolved within a wk
2010	Hispanic	Female	60 yr	Takotsubo cardiomyopathy	Emotional stress	acetylcholine receptor binding antibody	normal	Relieved within 10 mo
2023	Canada	Male	54 yr	Myocarditis	/	acetylcholine receptor antibody	Thymoma	Relieved within 2 wk
2013	France	Male	52 yr	Myocardial infarction (acute myocardial infarction)	Rituximab	seronegative	Thymus hyperplasia	Improvement
2022	USA	Male	66 yr	Takotsubo Cardiomyopathy	/	Anti acetylcholine receptor antibody	Normal	Partial improvement
2020	USA	Female	88 yr	Myocardial Infarction (acute myocardial infarction)	Pyridostigmine	/	/	improvement
2020	Japan	Female	87 yr	Coronary spastic angina	Intravenous immunoglobulin	AChR antibody	/	Rapidly relieved
2021	Netherlands	Male	81 yr	Takotsubo cardiomyopathy	Myasthenic crisis	/	/	Death
2009	Israel	Female	76 yr	Non-ST Elevation Myocardial Infraction	Intravenous immunoglobulins	/	/	Relieved within 10 d
2005	Brazil	Male	64 yr	Takotsubo cardiomyopathy	Acute respiratory failure	/	/	Complete recovery
2014	USA	Female	42 yr	Stress-induced cardiomyopathy (stress cardiomyopathy)	Chemotherapy	/	Thymoma	Partial improvement
2020	Japan	Female	66 yr	Right heart failure (heart failure)	/	Anti-titin antibody, anti-Kv1.4 antibody	/	Gradually improved
2020	USA	Male	61 yr	Cardiac arrest	Upper respiratory infection	Anti-Kv1.4 antibody		Fully recovered
2020	Japan	Female	45 yr	Right heart failure (heart failure)	Unknown etiology	Anti-MuSK antibody	/	Relieved within 10 mo
2012	Spanish	Female	83 yr	Takotsubo syndrome	/	Acetylcholine anti-receptor antibodyy	/	Partial improvement
2011	USA	Male	77 yr	Stress-induced non-ischemic cardiomyopathy	Plasmapheresis treatment	/	Normal	Recovered gradually over the 4 mo
2024	Japan	Female	51 yr	Takotsubo cardiomyopathy	Adrenalectomy	AChR antibody	Thymoma	Relieved within 72 d
2024	Japan	Male	78 yr	Myocarditis	Immune checkpoint inhibitors	/	/	Successful recovery
2019	Singapore	Female	59 yr	Takotsubo cardiomyopathy	Unknown etiology	AChR antibody	/	Partial improvement
2022	Spanish	Female	70 yr	Takotsubo cardiomyopathy	Pyridostigmine was withheld	/	Thymoma	Relieved within 14 d
2022	USA	Male	72 yr	Takotsubo cardiomyopathy	Plasmapheresis	/	/	Death
2024	Nepal	Female	31 yr	Takotsubo cardiomyopathy	Stressful events	AChR antibody	/	Remarkable recovery
2023	Italy	Female	57 yr	Takotsubo cardiomyopathy	Plasma exchange	AChR antibody	/	Relieved within 32 d
2015	UK	Male	70 yr	Takotsubo cardiomyopathy	Stress	AChR antibody	Normal	Relieved within 3 mo
2023	USA	Female	77 yr	Takotsubo cardiomyopathy	Acute hypoxia	/	Normal	Relieved within 7 d
2019	Thailand	Female	67 yr	Takotsubo cardiomyopathy	Respiratory tract infection	/	/	Relieved within 3 mo
2022	Austria	Female	78 yr	Takotsubo syndrome	Respiratory infection	/	/	Relieved within 10 d
2009	India	Female	40 yr	Tako-tsubo cardiomyopathy	Unknown etiology	/	Thymoma	Death
2017	USA	Female	69 yr	Takotsubo cardiomyopathy	Hyperthyroidism and methimazole	AChR antibody	/	Relieved within 4 wk
2015	China	Female	45 yr	Stress cardiomyopathy	Overworked		Thymoma	Relieved within 1 mo
2012	China	Female	64 yr	Takotsubo cardiomyopathy	Myasthenic crisis	/	/	Relieved within 3 mo
2011	France	Female	82 yr	Tako-Tsubo syndrome	Immunoglobulin	/	/	improvement
2021	USA	Male	68 yr	Myocarditis	Nivolumab	Negative	/	hospice care
2024	USA	Male	75 yr	Myocarditis	Pembrolizumab	AChR antibody	/	hospice care
2023	China	Male	55 yr	Myocarditis	PD-1 antibody	Negative	Thymoma	Relieved within 7 wk
2020	USA	Female	70 yr	Myocarditis	Pembrolizumab	AChR antibody	Thymic cancer	Relieved within 50 d
2021	China	Male	77 yr	Myocarditis	PD-1 antibody	AChR antibody, Titin antibody	/	Relieved within 2 mo
2022	China	Female	71 yr	Myocarditis	PD-1 inhibitor	AChR antibody	/	Relieved within 2 wk
2024	China	Male	72 yr	Myocarditis	PD-1 inhibitor	/	/	Relieved within 1 mo
2024	USA	Male	76 yr	Myocarditis	Nivolumab and ipilimumab	AChR antibody	/	Recovery
2020	Japan	Male	76 yr	Myocarditis	Pembrolizumab	AChR antibody	/	Death
2021	Spain	Female	48 yr	Myocarditis	Pembrolizumab	AChR antibody	Thymoma	Death
2022	China	Male	69 yr	Myocarditis	Camrelizumab	AChR antibody	/	Relieved within 2 mo
2019	USA	Female	78 yr	Myocarditis	Ipilimumab-nivolumab	Anti-striational antibody	/	Hospice care
2022	China	Male	65 yr	Myocarditis	Tislelizumab	AChR antibody, anti-titin antibody	/	Relieved within 3 mo
2020	China	Male	66 yr	Myocarditis	Sintilimab	AChR antibody	Thymoma	Relieved within 3 mo
2021	USA	Female	55 yr	Myocarditis	Abatacept	AChR antibody, anti-striated muscle antibody	/	Relieved within 32 d
2017	Japan	Female	69 yr	Myocarditis	Nivolumab	AChR antibody	/	Relieved within 36 d
2020	USA	Male	86 yr	Myocarditis	Cemiplimab	AChR antibody, anti-striated muscle antibody	/	Death
2013	Japan	Female	80 yr	Myocarditis	Pneumonia	AChR antibody	Thymoma	Death
2024	UK	Female	66 yr	Myocarditis	PD-1 inhibitor	AChR antibody, GAD antibody	/	Relieved within 12 d
2021	China	Female	47 yr	Myocarditis	Toripalimab	AChR antibody, anti-ryanodine receptor antibody	Thymoma	Relieved within 3 mo
2024	China	Female	82 yr	Myocarditis	Anti-PD-1 mAb	anti-titin antibody	/	Death
2024	Japan	Male	83 yr	Myocarditis	Atezolizumab	AChR antibody, nti-striated muscle antibody, anti-Kv1.4 antibody, anti-titin antibody	Normal	Relieved within 5 mo
2019	China	Male	69 yr	Myocarditis	/	AChR antibody, anti-titin antibody, anti-M7, SMA-Ab, CAE-Ab	Normal	Relieved within 2 wk
2023	USA	Male	67 yr	Myocarditis	Nivolumab	AChR antibody	/	Death
2024	USA	Male	80 yr	Myocarditis	Immune checkpoint inhibitor	AChR antibody	/	Death
2024	USA	Male	69 yr	Myocarditis	Pembrolizumab	Anti-LRP4	/	Partial improvement
2022	Japan	Female	55 yr	Myocarditis	Nivolumab	AChR antibody	Thymoma	Relieved within 54 d
2021	China	Male	77 yr	Myocarditis	PD-1 antibody	AChR antibody, anti-titin antibody	/	Relieved within 4 wk
2021	China	Female	69 yr	Myocarditis	PD-1 antibody	Anti-Ryanodine-Receptor-Ab, anti-Titin-Ab	/	Death
2020	Australia	Male	55 yr	Myocarditis	Nivolumab, ipilimumab	/	Normal	Relieved within 2 wk
2023	China	Female	45 yr	Myocarditis	Sintilimab	AChR antibody	Thymoma	Death
2012	Japan	Female	40 yr	myocarditis	Carboplatin, paclitaxel	AChR antibody	Thymoma	Death
1986	Netherlands	Male	48 yr	Myocarditis, intractable ventricular tachycardia	Respiratory infection	/	Thymoma	Death
2022	China	Male	42 yr	Myocarditis	Gemcitabine, carboplatin, sintilimab	/	Thymoma	Death
2022	China	Male	52 yr	Myocarditis	Docetaxel, cisplatin, tislelizumab	/	Thymoma	Thymoma
2001	Japan	Female	69 yr	Myocarditis	/	AChR antibody	Normal	Death
2006	France	Male	43 yr	Myocarditis	Thymoma resection	/	Thymoma	Death
2013	Japan	Male	31 yr	Myocarditis	Prednisolone dose tapering to 25 mg	/	Thymoma	Relieved within 4 d
2022	Thailand	Female	40 yr	Coronary artery spasm (Coronary spastic angina)	Pyridostigmine	AChR antibody	Normal	Relieved within 20 min
2023	Taiwan	Male	61 yr	Coronary artery spasm (coronary spastic angina), Intraoperative cardiogenic shock (cardiogenic shock)	Thymectomy	AChR antibody	Thymoma	Death
2013	India	Female	70 yr	Angina (coronary spastic angina)	/	AChR antibody	/	Relieved within 12 d
2024	Japan	Male	80 yr	Takotsubo syndrome	/	AChR antibody	Normal	Transfer to another hospital

AChR = acetylcholine receptor, GAD = glutamic acid decarboxylase, MG = myasthenia gravis, MuSK = muscle specific kinase.

**Figure 2.**
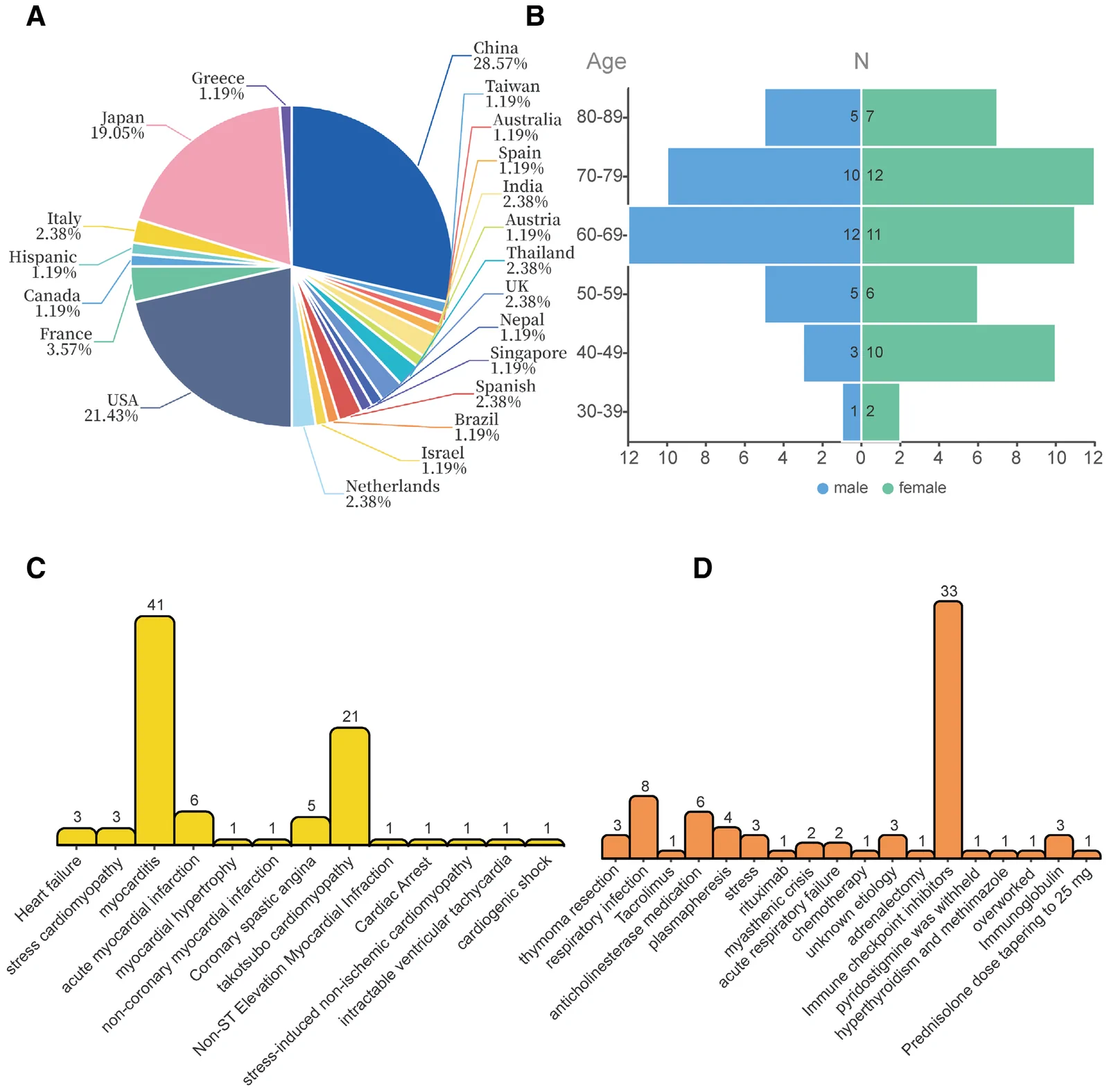
Clinical characteristics of MG patients with concurrent myocardial damage. (A) Case distribution by source; (B) gender distribution trends; (C) types of myocardial damage associated with MG; (D) etiological factors contributing to MG with myocardial damage. MG = myasthenia gravis.

### 3.2. Demographics and clinical characteristics

Our analysis comprised 84 patients exhibiting concurrent MG and myocardial damage, demonstrating a female predominance (57.1%, n = 48) over male patients (42.9%, n = 36), yielding a gender ratio of 1.3:1. The average age at disease onset was 64.5 years (range: 31–88 years). Notably, early-onset cases (≤39 years) accounted for merely 4.2% (n = 2/48) of female patients, whereas the majority of male patients (61.1%, n = 22/36) presented before 70 years of age (Fig. 2B).

Cardiac manifestations revealed distinct pathological patterns: nearly half of cases (48.8%, n = 41/84) presented with myocarditis, followed by takotsubo cardiomyopathy (25.0%, n = 21/84). Less frequent presentations included acute myocardial infarction (7.1%, n = 6/84) and coronary spastic angina (6.0%, n = 5/84), as illustrated in Figure 2C.

### 3.3. Etiological factors

Our analysis identified several key precipitating factors associated with myocardial impairment in this patient population. Immune checkpoint inhibitors emerged as the predominant risk factor (39.3%, 33/84), followed by respiratory infection (9.5%, 8/84) and administration of anticholinesterase medication (7.1%, 6/84). Other contributing elements included plasmapheresis (4.8%, 4/84), thymoma resection (3.6%, 3/84), stress (3.6%, 3/84), and intravenous immunoglobulin treatment (3.6%, 3/84).

Notably, the underlying cause remained undetermined in 3 cases (3.6%), while clinical documentation failed to specify triggering events in an additional 9 instances (10.7%), as illustrated in Figure 2D.

### 3.4. Immunological profile and thymic pathology

An important pathological feature of these patients involved disease-associated antibodies and thymic pathology. Among 52 patients with reported antibody status, 49 were seropositive, and 3 were seronegative. The data on antibody status were missing for 32 cases (38.1%, 32/84). The predominant pathogenic antibodies identified were Acetylcholine receptor (AChR) antibodies (80.8%, 42/52) and anti-titin antibodies (100.0%, 9/9). It is important to note that this finding is based on a very small subset of patients (n = 9) who underwent this specific test; therefore, this result must be interpreted with extreme caution and is not generalizable.

The thymic evaluation revealed 39 patients (46.4%, 39/84) had documented thymic evaluations; 13 patients (33.3%, 13/39) showed normal thymic morphology; 26 patients (66.7%, 26/39) exhibited thymic pathology, predominantly comprising thymic hyperplasia and thymoma. The data on thymic evaluation were missing for 45 cases (53.6%, 45/84).

### 3.5. Clinical outcomes

The study exhibited diverse clinical trajectories, with all patients demonstrating documented outcomes. Three patients (3.6%, 3/84) required hospice care, while 20 patients (23.8%, 20/84) culminated in mortality. Encouragingly, most patients (72.6%, n = 61) attained either complete remission or substantial clinical improvement, with recovery timelines varying significantly. Most responders showed resolution within 1 week to 3 months. A minority demonstrated recovery extending up to 10 months. Notably, several cases exhibited remarkably rapid clinical improvement. Collectively, our analysis shows a 72.6% survival rate alongside a 23.8% mortality rate for patients with MG complicated by myocardial damage.

### 3.6. Association between mortality and clinical parameters

In our final analytical assessment, we evaluated potential correlations between fatal outcomes and several key clinical parameters, including patterns of myocardial damage, precipitating factors, pathogenic autoantibodies, and thymic pathology. Analysis of fatal outcomes revealed distinct patterns of myocardial involvement, with myocarditis being the predominant manifestation (70.0%, 14/20), followed by Takotsubo cardiomyopathy (15.0%, 3/20; Fig. 3A). The primary identified triggers associated with these fatal outcomes included immune checkpoint inhibitors (50.0%, 10/20), anticholinesterase medications (10.0%, 2/20), respiratory infections (10.0%, 2/20), and thymoma resection (10.0%, 2/20; Fig. 3B). Immunological assessment identified pathogenic antibodies in 13 mortality cases, predominantly AChR antibodies (84.6%, 11/13) and anti-titin antibodies (23.1%, 3/13). Among the reported fatalities, 12 cases involved thymic pathology, with only 2 instances (16.7%, 2/12) exhibiting a structurally normal thymus. Thymic anomalies were identified in the remaining 10 cases (83.3%, 10/12), all diagnosed as thymomas. The comparison of clinical variables between fatal cases and non-fatal cases is shown in Table 3. Although there is no statistically significant difference in the clinical variables between fatal and non-fatal cases, this suggests that we should pay attention to these factors.

**Table 3:** Clinical variables for fatal and non-fatal cases.

	AChR antibody positivity	Thymoma	Myocarditis	Immune checkpoint inhibitor use	Acute myocardial infarction
Non-fatal cases (n)	31	14	27	23	5
Fatal cases (n)	11	10	14	10	1
**χ^2^‌**	2.326				
*P*	.687				

**Figure 3.**
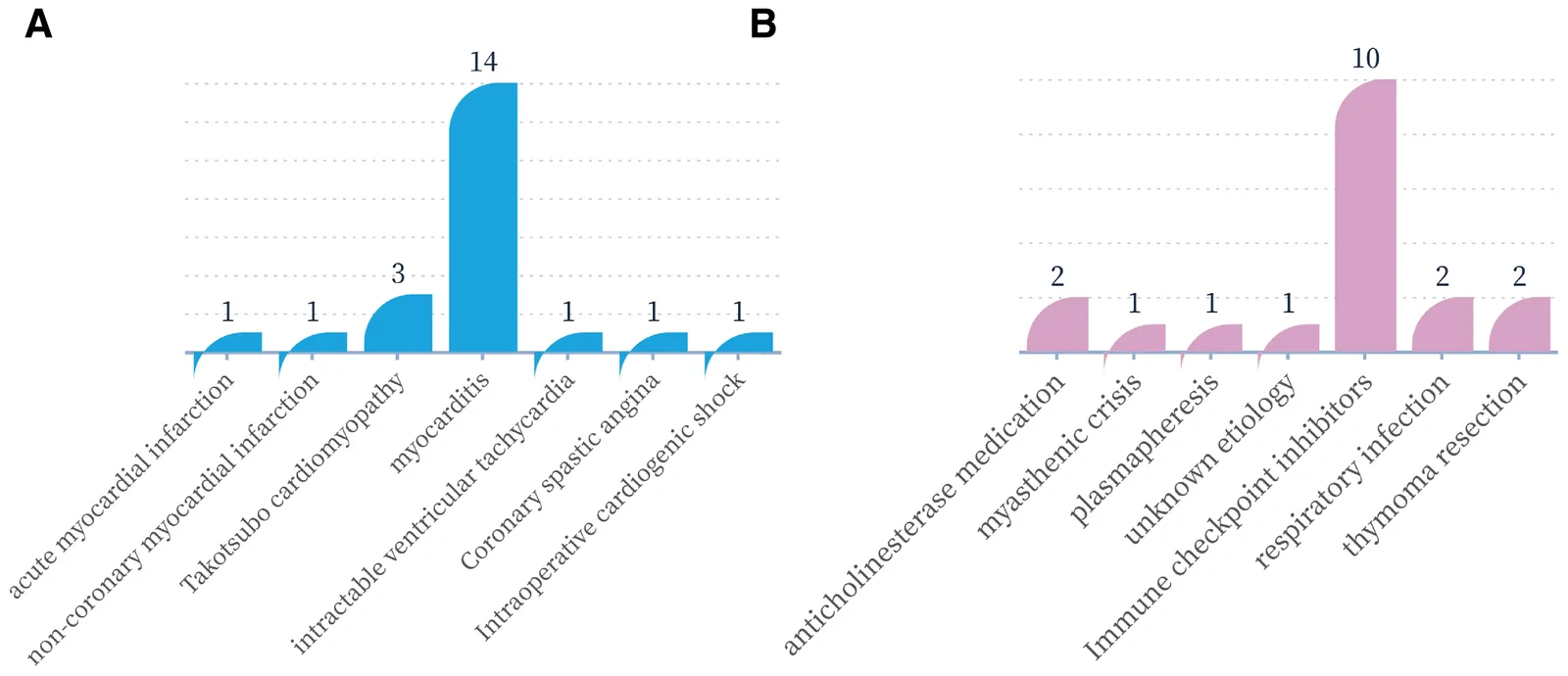
Clinical characteristics of fatal cases in MG patients with concurrent myocardial damage. (A) Classification of myocardial damage types observed in deadly cases; (B) identified etiological factors contributing to mortality. MG = myasthenia gravis.

Although the chi-square test revealed no statistically significant differences in the prevalence of AChR antibody positivity, thymoma, myocarditis, immune checkpoint inhibitor use, or acute myocardial infarction between the fatal and non-fatal groups (*P* > .05), these conditions presented with higher frequencies, suggesting potential clinical relevance that warrants further investigation.

## 4. Discussion

This retrospective study analyzed clinical data from 84 MG patients. The population demonstrated a slight female predominance, with a gender distribution of 1.3:1 (48 females to 36 males). Symptom onset occurred at a mean age of 64.5 years (range: 31–88). Notably, cardiac complications were observed in nearly half of the study (48.8%), with myocarditis representing the most frequent manifestation. Other cardiovascular presentations included takotsubo cardiomyopathy (25.0%), acute myocardial infarction (7.1%), and coronary spastic angina (6.0%). Identified precipitating factors encompassed immunomodulatory therapies (immune checkpoint inhibitors, immunoglobulin administration), medical interventions (plasmapheresis, thymoma resection), pharmacological agents (anticholinesterase drugs), infectious triggers (respiratory infections), and physiological stressors (stress). However, the causative factors remained undetermined in 3.6% of cases.

In this patient cohort, AChR antibodies were detected in 80.8% of cases, implying a possible link between AChR autoimmunity and the co-occurrence of MG with myocardial damage. Thymic data were available for only 46.4% (39/84) of patients, among whom 66.7% exhibited thymic abnormalities. Interestingly, 33.3% maintained normal thymic morphology. Nevertheless, these findings may not establish a definitive relationship between thymic status and cardiac involvement in myasthenia gravis, given that thymic evaluation was absent in most cases (53.6%).

In this study, all cases included prognostic data. Encouragingly, 72.6% of patients demonstrated favorable therapeutic responses. However, 3.6% required palliative care, while 23.8% succumbed to their condition. We subsequently investigated potential correlations between clinical variables and patient characteristics.

Among deceased patients, AChR antibodies predominated, reinforcing the crucial relationship between AChR autoimmunity and disease. Notably, thymic pathology was identified in 50.0% of fatal cases, with thymectomy emerging as a frequent contributing factor.

While thymic status remained undocumented in 53.6% of cases (a limitation requiring consideration), we postulate that thymic abnormalities may significantly influence clinical variables. Intriguingly, 2 mortality cases presented with normal thymic histology. Detailed analysis revealed these patients exhibited acute myocardial infarction and myocarditis. In comparison, the predominant cardiac manifestations among fatal cases were myocarditis and Takotsubo cardiomyopathy.

Although only 1 mortality resulted from acute myocardial infarction, this finding underscores the profound impact of acute coronary events on patient survival. Furthermore, the predominant cardiac pathologies linked to mortality were myocarditis (70.0%) and Takotsubo cardiomyopathy (15.0%). The primary triggering factors included immune checkpoint inhibitors (50.0%), anticholinesterase drugs (10.0%), respiratory infections (10.0%), and thymectomy (10.0%). Among the 13 fatal cases with documented autoantibodies, AChR antibodies predominated (84.6%), followed by anti-titin antibodies (23.1%).

The thymic evaluation was performed in 12 cases, revealing standard thymic architecture in 2 instances (16.7%). Pathological thymic alterations were identified in 10 cases (83.3%), all of which demonstrated thymomatous changes. Notably, the 2 fatalities with normal thymic morphology exhibited concurrent myocarditis and acute myocardial infarction. In fatal cases, we observed more frequent myocardial involvement, thymus pathology, antibodies, and immune checkpoint inhibitors. Although these differences did not reach statistical significance compared with non-fatal cases, this finding still warrants further exploration in larger-scale, more rigorous studies.

It is important to note that while the chi-square test did not yield statistically significant results for these variables, we elected to discuss them because of their well-established pathophysiological roles in the literature. The lack of statistical significance is likely attributable to the relatively small sample size, rather than a complete absence of clinical effect. Therefore, these factors are presented as observed trends rather than definitive prognostic indicators.

Our results indicate that the pathophysiological mechanisms influencing clinical outcomes in myasthenia gravis patients with concomitant myocardial damage may involve multiple interrelated factors. These include AChR antibody-mediated autoimmunity, thymomatous pathology, inflammatory myocardial involvement (particularly myocarditis), immune checkpoint inhibitor therapy, and acute ischemic cardiac events. The complex interplay between these elements likely contributes to the observed prognostic variations in this patient population.

## 5. Conclusion and limitation

While our investigation provides valuable insights, several methodological constraints should be acknowledged. The study’s observational design, incorporating retrospective and prospective elements, inherently limits causal inferences. Although we analyzed a substantial cohort of 84 myasthenia gravis cases complicated by myocardial damage, potential selection biases must be considered due to incomplete clinical documentation regarding precipitating factors, pathogenic autoantibodies, and thymic status in some instances.

Notwithstanding these limitations, our dataset represents a comprehensive and rigorously validated collection of cases. The present analysis offers a detailed examination of clinical characteristics and explores plausible pathophysiological mechanisms underlying the association between myasthenia gravis and cardiac involvement.

This investigation represents the first comprehensive synthesis of clinical characteristics in MG patients with concomitant myocardial damage reported in contemporary literature. Our analysis revealed myocarditis as the predominant cardiac manifestation in this patient population. In more than half of the cases (80.8%), anti-AChR antibodies were detected. While specific therapeutic regimens were not systematically documented, a substantial proportion of patients (72.6%) demonstrated significant clinical improvement.

In conclusion, our findings highlight several clinically relevant variables, such as myocardial involvement and thymic pathology, that were observed more frequently in fatal cases. These observations should be considered hypothesis-generating and merit further investigation in larger, prospective cohort studies to clarify their potential role in the disease course.

## Author contributions

**Conceptualization:** Chaofang Lei.

**Methodology:** Chaofang Lei, Le Xie.

**Data curation:** Zhigang Chen.

**Investigation:** Jiaxu Chen.

**Supervision:** Junlin Jiang.

**Writing – original draft:** Chaofang Lei.

**Writing – review & editing:** Chaofang Lei, Dahua Wu, Junlin Jiang.
